# Dose equivalency and efficacy of biosimilar erythropoietin stimulating agents: Data from real clinical practice

**DOI:** 10.1002/prp2.594

**Published:** 2020-06-10

**Authors:** Abduzhappar Gaipov, Alpamys Issanov, Zhanar Mursalova, Nazia Tulegenova, Zoya Kakim, Mukhit Baizakov, Saltanat Tuganbekova, Mohamad Aljofan

**Affiliations:** ^1^ Department of Clinical Sciences Nazarbayev University School of Medicine Nur‐Sultan Kazakhstan; ^2^ Department of Dialysis Diaverum Kazakhstan Almaty Kazakhstan; ^3^ Department of Dialysis Diaverum Kazakhstan Temirtau Kazakhstan; ^4^ Department of Dialysis Hemodialysis Center Daru Aktobe Kazakhstan; ^5^ Department of Internal Medicine National Scientific Medical Center Nur‐Sultan Kazakhstan; ^6^ Department of Biomedical Sciences Nazarbayev University School of Medicine Nur‐Sultan Kazakhstan

**Keywords:** biosimilar, Kazakhstan, originator, renal anemia, switching

## Abstract

Recently, biosimilar erythropoietin stimulating agents become available in Kazakhstan. Important properties of the biosimilar such as dose equivalency to the original medicine (originator) and the ability to maintain hemoglobin target levels remain insufficiently described in many clinical settings. Thus, the current study aims to determine dose equivalency and hemoglobin target levels in a cohort of dialysis patients who were switched from the originator to biosimilar. Retrospective data of 74 patients from different dialysis centers who received at least 6 months of originator and switched to biosimilar and had at least 6 months follow‐up were analyzed. The clinical data of 32 male and 42 female patients were collected. The mean age was 52.5 ± 13.5 years. There is no significant difference in mean levels of hemoglobin during pre‐switching from originator to biosimilar (6 months prior) and post switching period (9 months after). Additionally, a subgroup analysis of 59 patients who received originator (epoetin beta), 6 months before the switch, showed similar level of hemoglobin (110.7 ± 14 vs 113.2 ± 10 g/L, *P* = .05) 6 months after the switch to biosimilar (epoetin zeta) at the equivalent dose regimen (69.5 ± 29 vs 68.1 ± 30 IU/kg/wk, *P* = .55). However, after 9 months of switching, patients using lower doses of biosimilar (69.5 ± 29 vs 63.3 ± 30 IU/kg/wk, *P* < .01), showed significantly higher levels of hemoglobin (110.7 ± 14 vs 114.7 ± 8 g/L, *P* = .01) compared to preswitching period. In conclusion, long‐term use of lower doses of biosimilar managed to maintain hemoglobin within the target levels.

## 1 INTRODUCTION

Renal anemia, a common complication of end stage renal disease (ESRD) is associated with high morbidity and mortality and thought to occur in over 90% of patients receiving renal replacement therapy.^1^, ^2^ The vast majority, if not all patients with renal anemia require maintenance replacement therapy with human recombinants erythropoietin stimulating agents (rESA) such as epoetin, which is a recombinant form of erythropoietin, a hematologic growth factor that induces proliferation and maturation of red blood cells and is used in the treatment of anemia caused by renal disease.^3^ rESA treatments were shown to raise hemoglobin and hematocrit levels in patients with end stage renal disease on hemodialysis.^3^

Despite their proven efficacy and crucial role in the treatment of renal anemia, the high cost of rESA agents is a contributing factor to the increasing cost of treatment, makes them inaccessible to many patients. An alternative option to the use of biologic agents (originator) and a more cost effective choice is the use of biosimilar agents, which have similar, but not identical molecular shape, efficacy, and safety to the original product.^4^ They are being widely developed to create price competition and cheaper alternatives to the costly originators.

The lower costs of biosimilars will respite some of the financial pressure on healthcare budgets and allow greater access for patients. This would also provide major advantages for patients and communities such as that financial resources may be more efficiently allocated to other important uses, which makes more innovative therapies available to patients.^5^ In addition to being a cost saving phenomenon, switching to biosimlars is something to be considered as a medical issue, particularly if it could occur with other medicines as well.^6^

Since the year 2017, biosimilar ESA (bESA) have become available in Kazakhstani trade. Due to their lower prices compared to originator (rESA), a few of the country's dialysis centers switched their patients to bESA. However, important properties of bESA such as dose equivalency to rESA and their ability to maintain hemoglobin (HB) target levels remain unknown, which might have contributed to low switching rate in Kazakhstan. Hence, determining these important properties will increase the enthusiasm toward biosimilar and would encourage many clinicians to switch their patients to a more cost‐effective alternative. Therefore, the aim of the current study is to determine the dose equivalency and HB target level in a cohort of dialysis patients who were switched from rESA (originator) to bESA (biosimilar) during real clinical practice.

## 2 MATERIALS AND METHODS

### 2.1 Study population

De‐identified data without personal information were extracted from dialysis electronic medical information system (Diaverum IRIMS ‐ International Renal Information Management System) including longitudinal laboratory and treatment variables for patients treated between January 2017 and July 2018. Inclusion criteria: End‐stage renal disease patients, who were on maintenance hemodialysis, received rESA for at least 6 months and switched to bESA with a minimum record of 6 months follow‐up. Exclusion: patients other than those with renal anemia, patients who did not switch from originator to biosimilar, and those who do not meet the minimum time limits for treatment and follow‐ups were excluded.

### 2.2 Study design and setting

The current study is designed as a retrospective study that included patients from different dialysis centers in three different regions of Kazakhstan (Aktobe, Temirtau, and Almaty regions). In order to get meaningful data, patients with at least 1 year of health assistance prior to the baseline date were selected. Baseline data are defined as data that were collected prior to the switch. Demographic and clinical characteristics of the cohort at the baseline were retrieved. Specifically, age, gender, factors related to anemia such as body mass index (BMI), serum iron level, hemodialysis vintage, and ferritin levels were collected and analyzed. The data were analyzed to determine the long‐term effect of bESA on dose equivalency and HB target level. The mean ESA dose expressed in IU/kg/wk and the mean of HB level expressed in g/L were calculated to compare the data, obtained 6 months prior to switching and 6 months after switching from originator to biosimilar. Additional 3 months period were analyzed to determine the long‐term effect of biosimilar on dose equivalency and hemoglobin target level.

### 2.3 Statistical analysis

Data were extracted form electronic medical information system on a Microsoft Office Excel^®^ (2010) spreadsheet and transferred to STATA MP Version 15 (STATA Corporation). Mean ± SD for normal and median—interquartile range (IQR) for non‐normal distribution of numerical variables were calculated for all entries. Categorical variables were presented as frequencies and relative frequencies in percentages. Comparison of repeated two or more measurements of HB and ESA at different time points were assessed with paired t‐test or Wilcoxon signed rank test and Friedman’s test with post‐hoc non‐parametric paired tests where applicable. Data were presented as a tables and graphical figures to better describe the study design and results. Two sided *P <* .05 were considered significant.

## 3 RESULTS

### 3.1 Demographic data

In total, we included 74 dialysis patients who received at least 6 months of rESA and switched to bESA (epoetin zeta). The cohort is made up of 42 (56.8%) female patients and 32 (43.2%) males with mean age of 52.5 ± 13.5 years and average BMI of 67.5 ± 17.8 kg/m^2^ (Table 1). The mean level HB at the time of switching was (110.8 ± 14.8) (Table 2). The included cohort was made up of 59 patients who received epoetin beta, 12 patients who received the long acting ESA (methoxy‐polyethylene‐glycol epoetin beta), and 3 patients who were receiving another long acting ESA (darbepoetin alfa), before switching to bESA (epoetin zeta). There were two follow‐up periods a 6 months period and a 9 months follow‐up period.

**Table 1:** Baseline characteristics of study group

Demographics	n = 74
Age, y	52.5 ± 13.5
Gender, female (%)	42 (56.8%)
BMI, kg/m^2^	67.5 ± 17.8
KT/V	1.5 ± 0.16
HD vintage, mo	36.0 (18.9‐56.3)
Ferritin, µg/L	1143.8 (904.6‐1791.5)
Serum Fe, µmol/L	11.5 ± 3.9

Note

Data presented as Mean ± SD or Median (IQR) as appropriate

Abbreviations: BMI, body mass index; HD, hemodialysis; KT/V, dialysis adequacy.

**Table 2:** Mean HB levels before and after the switching from rESA to bESA (n = 74)

Months, before switching	HB level, g/L	HB < 100 g/L^a^	Months, after switching	HB level, g/L	HB < 100 g/L^a^
−6 mo	109.2 ± 14.1	10 (13.5%)	+1 mo	110.0 ± 11.9	11 (14.8%)
−5 mo	109.2 ± 15.4	10 (13.5%)	+2 mo	111.9 ± 13.6	9 (12.1%)
−4 mo	109.5 ± 16.0	14 (18.9%)	+3 mo	114.2 ± 11.8	6 (8.1%)
−3 mo	113.0 ± 13.3	8 (10.8%)	+4 mo	113.3 ± 10.1	7 (9.4%)
−2 mo	113.4 ± 15.9	9 (12.1%)	+5 mo	112.6 ± 12.4	10 (13.5%)
−1 mo	110.8 ± 14.8	13 (17.5%)	+6 mo	113.5 ± 12.1	8 (10.8%)
—	—	—	+7 mo	114.6 ± 11.3	6 (8.1%)
—	—	—	+8 mo	111.8 ± 10.9	7 (9.4%)
—	—	—	+9 mo	113.7 ± 9.9	7 (9.4%)

Note

Abbreviations: HB, hemoglobin.

^a^ Number of patients with HB < 100 g/L (who not reaching the target level)

### 3.2 Hemoglobin target level

The patient’s mean level HB 6 months before the switching from rESA to bESA is compared to that of the 6 and 9 months periods following switching. The results show no significant difference in mean levels of HB during the pre‐ and post conversation periods (Table 2). Notably, the results showed that 14.4% of patients were under the HB target level during the last 6 months prior to switching from rESA to bESA. Interestingly, this figure was reduced to 11.5% at 6 months after switching from rESA to bESA and continued to decrease further to 9.0% at nine months after switching.

### 3.3 Dose equivalency

**Figure 1:**
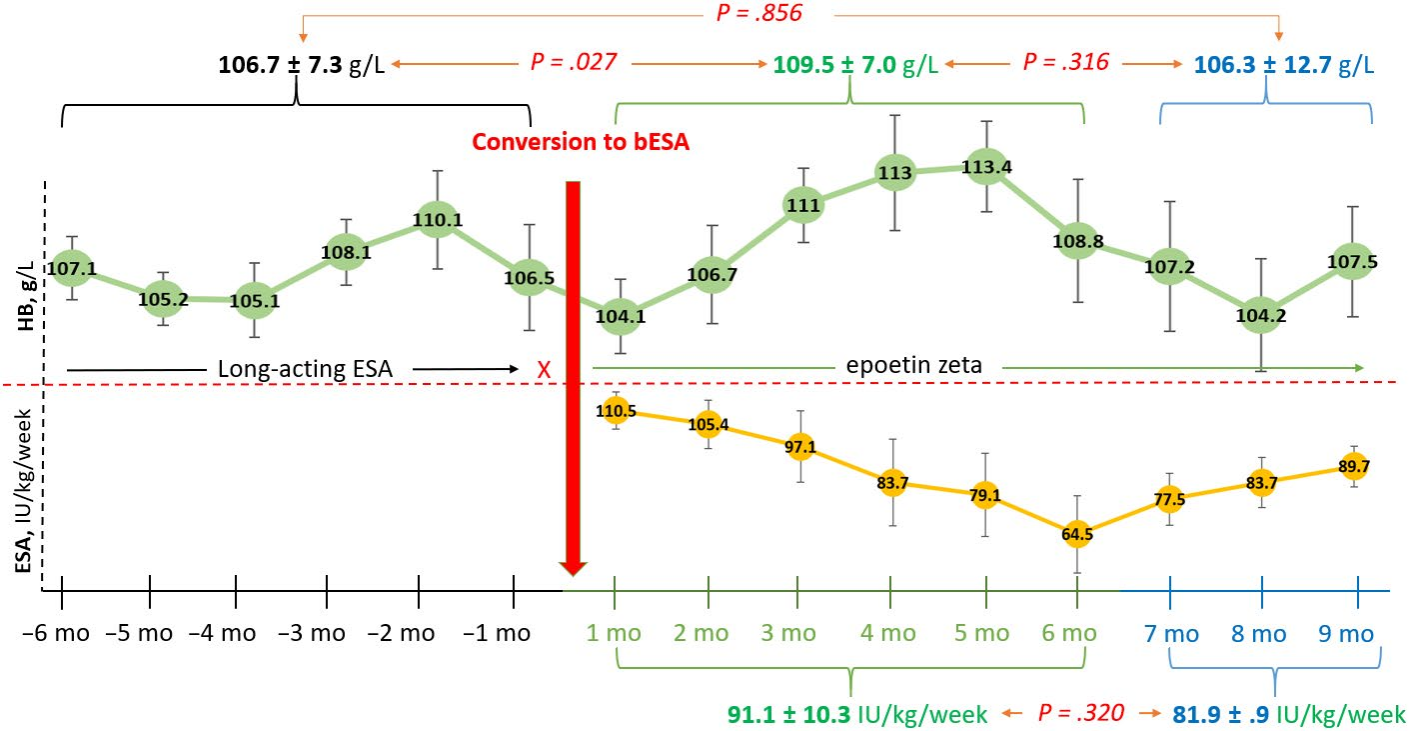
ESA dose and HB levels before and after the switching from long acting ESA (methoxy‐polyethylene‐glycol epoetin beta and darbepoetin alfa) to epoetin zeta (n = 15). ESA, erythropoietin stimulating agents; bESA, biosimilar ESA; HB, hemoglobin; mo, months

A subgroup analyses comparing the HB levels, of 59 patients who were receiving epoetin beta, and those patients who were receiving the long acting ESA (methoxy‐polyethylene‐glycol epoetin beta and darbepoetin alfa) 6 months before switching to the results of 6 and 9 months after the switching to epoetin zeta were carried out. The HB levels of patients who were receiving the long acting ESA 6 months before switching remained the same as to 6 months (106.7 ± 7.3 vs 109.5 ± 7 g/L, *P* = .03) and 9 months (106.7 ± 7.3 vs 106.3 ± 12.7 g/L, *P* = .32) after switching to bESA at an equal dose regimen (91.1 ± 10.3 vs 81.9 ± 0.9 IU/kg/wk, *P* = .32) (Figure 1).

**Figure 2:**
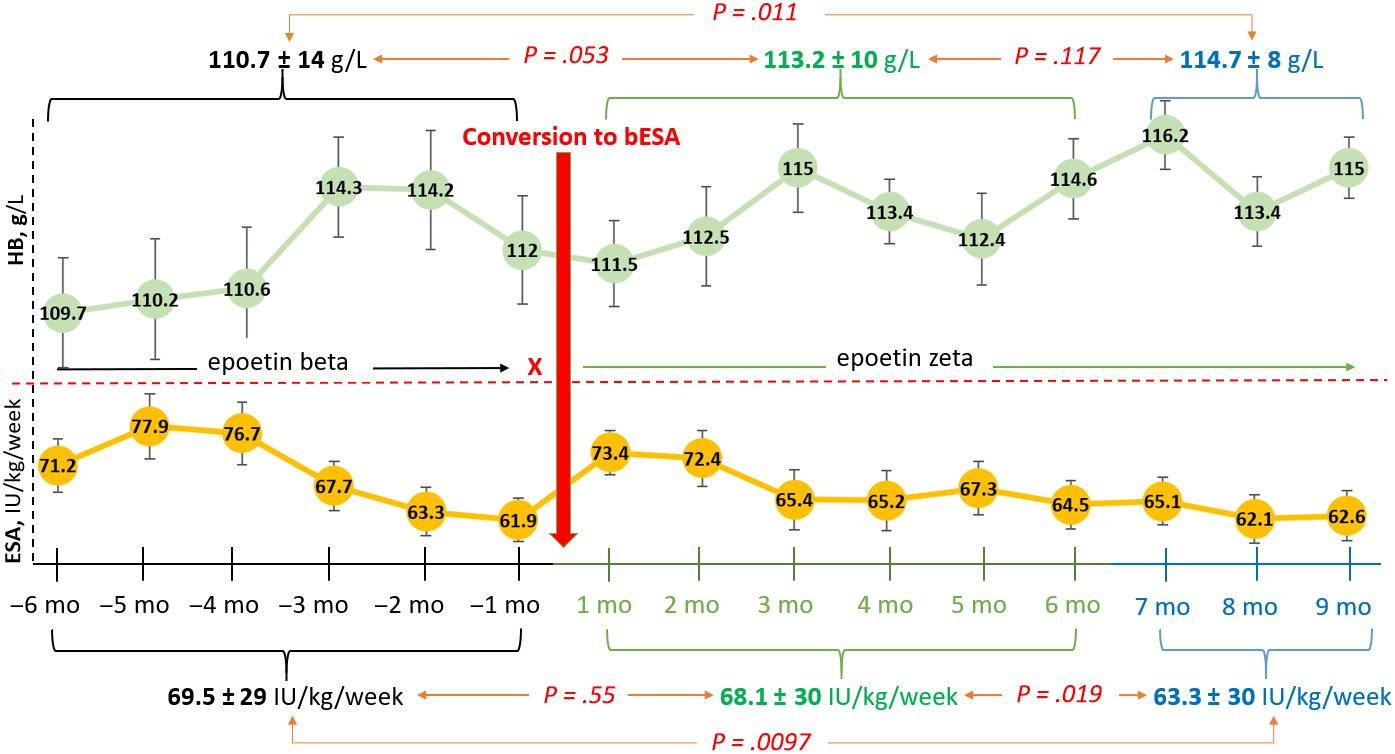
ESA dose and HB levels before and after switching from epoetin beta to epoetin zeta (n = 59). ESA, erythropoietin stimulating agents; bESA, biosimilar ESA; HB, hemoglobin; mo, months

In addition, the results showed that patients receiving epoetin beta during the last 6 months, had maintained similar level of HB (110.7 ± 14 vs 113.2 ± 10 g/L, *P* = .05) to that after conversation to epoetin zeta during the following 6 months, at the equivalent dose regimen (69.5 ± 29 vs 68.1 ± 30 IU/kg/wk, *P* = .55) (Figure 2). However, during the 7th, 8th, and 9th months after switching, patients switched to epoetin zeta were shown to have significantly higher levels of HB (110.7 ± 14 vs 114.7 ± 8 g/L, *P* = .01) on lower dose of bESA compared to preswitching period (69.5 ± 29 vs 63.3 ± 30 IU/kg/wk, *P* < .01) (Figure 2).

## 4 DISCUSSION

To our knowledge, this is the first study from Kazakhstan that intended at evaluating the impact of switching from rESA to bESA on patients with renal anemia from the real clinical setting using hard clinical outcomes. The data analyses of 74 dialysis patients who switched from rESA to bESA showed no difference in efficacy on maintain HB target levels and dose equivalency. The results also showed that the long‐term use of lower doses of bESA maintained HB within the target levels.

In addition to the high mortality and morbidity rates, patients with renal anemia are at an increased risk of cardiovascular diseases and hospitalization compared with chronic kidney disease (CKD) patients without anemia.^7^, ^8^ Evidence show that the direct healthcare costs of CKD patients with anemia are significantly higher than patients without anemia.^9^ Therefore, providing alternative or cheaper therapies than the originators would likely reduce the overall costs. Thus, biosimilars represent a cost effective alternative that could contribute to the economic sustainability of health systems.^10^ Biosimilar agents are biological drugs that are approved via strictly defined regulatory pathways on the basis that their efficacy, safety and quality are comparable to the originator.^11^

Biosimilars are widely used in many different countries with the European Union (EU) countries leading the way, where the first biosimilar was approved in 2006 and currently more than 40 biosimilars including bESA are available in EU market.^12^ Other countries out of the EU started the registration and use of biosimilars much later than the EU. For instance in the USA, the FDA approved the clinical use of biosimilar ESA in 2018, which is 12 years after the EU.^13^ It is likely that the approval came after the publication of the randomized clinical trial by Fishbane et al that described the efficacy of the ESA biosimilar.^14^ However, Kazakhstan was the first country among the former Soviet member countries to register and approve the use of biosimilar epoetin in 2017. It is likely that the use of bESA will soon be widely used in many countries in the central Asia region and the middle east.

Despite the proven comparability of the efficacy and safety of biosimilar to the originator medicine and the great opportunity for cost saving, the acceptance of biosimilar agents among the medical community remains limited in some countries and therapeutic areas.^6^ There are several cited reasons that could explain the lack of enthusiasm toward biosimilars, including the lack of provider confidence, uncertainty about substitution, and a lack of patient awareness and education.^12^

In Kazakhstan, bESA become available in 2017, after which a few dialysis centers switched their patients to the newly introduced alternative. While it is not surprising that some clinicians remain cautious about the switch, the current results aim to serve as a proof of concept from real clinical data that bESA are as effective as their originators in keeping the HB level within target level with an equivalent dose. In fact, the results showed that long‐term use of low bESA doses were capable of maintaining HB at target levels. Studies based on real‐clinical data, including the present study have provided strong evidence that rESA are an effective and well‐tolerated option for the treatment of renal anemia. However, as the experience and evidence with bESA increase, many of the clinician’s cautions and concerns regarding bESA would eventually be improved.

The current study has some limitations including the relatively small sample size of the studied population, which is insufficient to demonstrate statistical significance between the pre‐ and post switching periods. The second limitation is that the study did not investigate the safety and side effects of the biosimilar, however, the records did not show any reports of yellow cards or complains during the treatment periods. Another imitation is due to the study design. The current study is a retrospective study from real clinical practice with no proposed research design, therefore, the results cannot be considered as randomized clinical trials. This study did not collect information on confounding factors that could potentially influence HB levels when patients switched from bESA to rESA. Also, we did not compare the findings with the same reference originator, epoetin alfa, as in Kazakhstan and at the time of this study, no patients were treated with epoetin alfa. Finally, a possible selection bias may have occurred among the selected cohort who switched to the bESA, but did not complete the 6 months period or stopped due to side effects or complications. However, there are no reports of patient dropouts or treatment discontinuation during the studied period.

### 4.1 Conclusion

The results of this retrospective cohort from real clinical practice, showed no difference between biosimilar ESA compared to recombinant ESA on dose equivalency and maintaining HB target level in dialysis patients. At the long‐term period, there was a trend to maintain the target level of HB even at lower dose of biosimilar ESA compared to the originator ESA. This finding can increase the confidence in biosimilar as an effective alternative to the originator ESA in the treatment of renal anemia and would improve significant cost savings and patient access.

## CONFLICT OF INTEREST

The authors declare no conflict of interest.

## AUTHOR CONTRIBUTIONS

AG, ZM, and MA contributed to the study design and initiation. NT, ZK, MB, and ST were involved in data collection and some data analyses. AI performed the statistical analyses and figure generation. AG and AI wrote the first draft of the manuscript. MA finalized the manuscript, which was subsequently approved by all authors. All authors read and approved the final manuscript.

## ETHICS STATEMENT

The study was approved by the Institutional Review Ethics Committee, and exempted from informed consent since it is a retrospective study.

## PRINCIPAL INVESTIGATOR STATEMENT

The authors confirm that the Principal Investigator for this paper is Abduzhappar Gaipov and that he had direct clinical responsibility for patients.

## Data Availability

The data that support the findings of this study are available from the corresponding author upon reasonable request.

## References

[1] Nakhoul G , Simon JF . Anemia of chronic kidney disease (vol 83, pg 613, 2016). Clev Clin J Med. 2016;83(10):739. 27726834

[2] Solak Y , Atalay H , Biyik Z , et al. Colchicine toxicity in end‐stage renal disease patients: a case‐control study. Am J Ther. 2014;21(6):e189‐e195. 22874645 10.1097/MJT.0b013e31825a364a

[3] LiverTox: Clinical and Research Information on Drug‐Induced Liver Injury [Internet]. Bethesda, MD: National Institute of Diabetes and Digestive and Kidney Diseases; 2012. Erythromycin. [Updated 2017 Aug 10]. 31643176

[4] Nixon NA , Hannouf MB , Verma S . The evolution of biosimilars in oncology, with a focus on trastuzumab. Curr Oncol. 2018;25(Suppl 1):S171‐S179. 29910660 10.3747/co.25.3942 PMC6001768

[5] Horbrand F , Bramlage P , Fischaleck J , Hasford J , Brunkhorst R . A population‐based study comparing biosimilar versus originator erythropoiesis‐stimulating agent consumption in 6,117 patients with renal anaemia. Eur J Clin Pharmacol. 2013;69(4):929‐936. 23052412 10.1007/s00228-012-1412-5

[6] Belleudi V , Trotta F , Addis A , et al. Effectiveness and safety of switching originator and biosimilar epoetins in patients with chronic kidney disease in a large‐scale Italian cohort study. Drug Saf. 2019;42(12):1437‐1447. 31228010 10.1007/s40264-019-00845-y PMC6858470

[7] Li S , Foley RN , Collins AJ . Anemia and cardiovascular disease, hospitalization, end stage renal disease, and death in older patients with chronic kidney disease. Int Urol Nephrol. 2005;37(2):395‐402. 16142575 10.1007/s11255-004-3068-2

[8] Eriksson D , Goldsmith D , Teitsson S , Jackson J , van Nooten F . Cross‐sectional survey in CKD patients across Europe describing the association between quality of life and anaemia. BMC Nephrol. 2016;17(1):97. 27460779 10.1186/s12882-016-0312-9 PMC4962379

[9] van Nooten FE , Green J , Brown R , Finkelstein FO , Wish J . Burden of illness for patients with non‐dialysis chronic kidney disease and anemia in the United States: review of the literature. J Med Econ. 2010;13(2):241‐256. 20438399 10.3111/13696998.2010.484307

[10] Stoppa G , D’Amore C , Conforti A , et al. Comparative safety of originator and biosimilar epoetin alfa drugs: an observational prospective multicenter study. BioDrugs. 2018;32(4):367‐375. 30030767 10.1007/s40259-018-0293-2 PMC6061296

[11] Goldsmith D , Dellanna F , Schiestl M , Krendyukov A , Combe C . Epoetin biosimilars in the treatment of renal anemia: what have we learned from a decade of European experience? Clin Drug Investig. 2018;38(6):481‐490. 10.1007/s40261-018-0637-1 PMC5951862 29500617

[12] Smeeding J , Malone DC , Ramchandani M , Stolshek B , Green L , Schneider P . Biosimilars: considerations for payers. P T. 2019;44(2):54‐63. 30766011 PMC6355057

[13] FDA Drug Administration website . FDA approves first epoetin alfabiosimilar for the treatment of anemia. https://www.fda.gov/news‐events/press‐announcements/fda‐approves‐first‐epoetin‐alfa‐biosimilar‐treatment‐anemia [press release]. Accessed May 15, 2018.

[14] Fishbane S , Singh B , Kumbhat S , Wisemandle WA , Martin NE . Intravenous epoetin alfa‐epbx versus epoetin alfa for treatment of anemia in end‐stage kidney disease. Clin J Am Soc Nephrol. 2018;13(8):1204‐1214. 29921734 10.2215/CJN.11631017 PMC6086700

[15] Gaipov A , Issanov A , Mursalova Z , et al. SUN‐275 dose equivalency and efficacy of biosimilar erythropoietin stimulating agents: data from real clinical practice. Kidney Int Rep. 2020;5(3):S313‐S314. 10.1002/prp2.594 PMC7287030 32524766

